# Supplementary material for: DEK promotes mammary hyperplasia and is associated with H3K27me3 epigenetic modifications
Source: Life Sci Alliance. 2025 Jun 25;8(9):e202503230. doi: 10.26508/lsa.202503230 (PMC12198979; doi:10.26508/lsa.202503230)

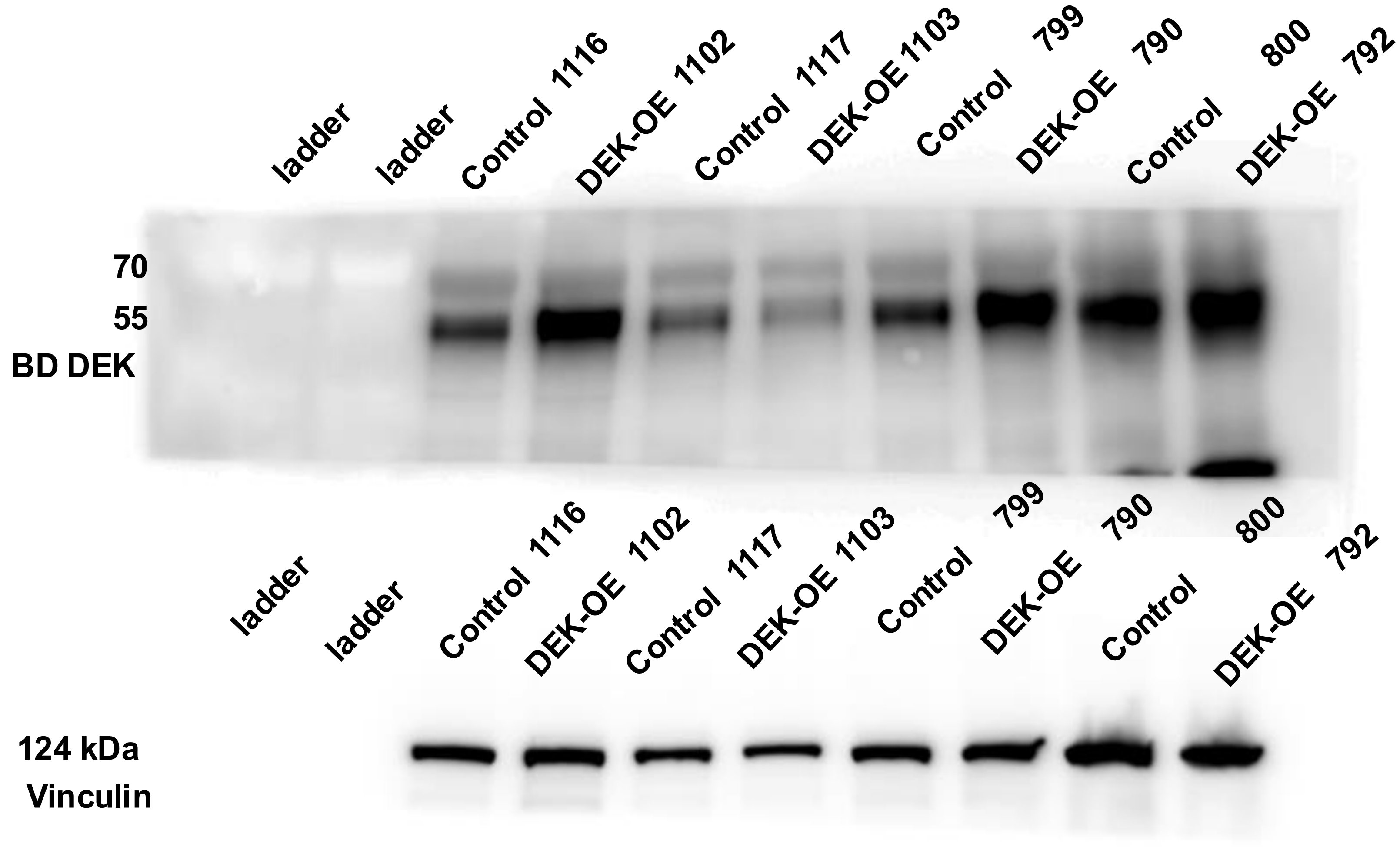

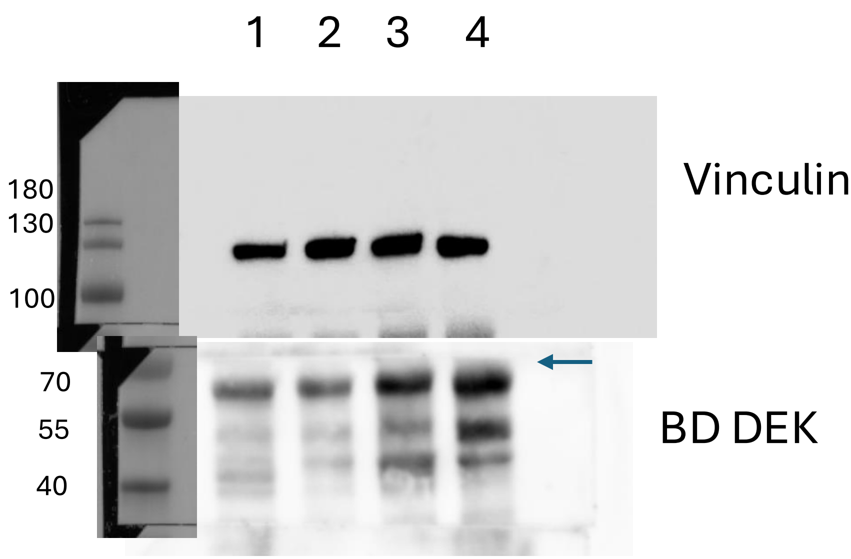

1. 1673 BiLDEK + tTa + +DOX (8 weeks)
2. 1674 BiLDEK + tTa + +DOX (8 weeks)
3. 1687 BiLDEK + tTa + (8 weeks)
4. 1688 BiLDEK + tTa (8 weeks)

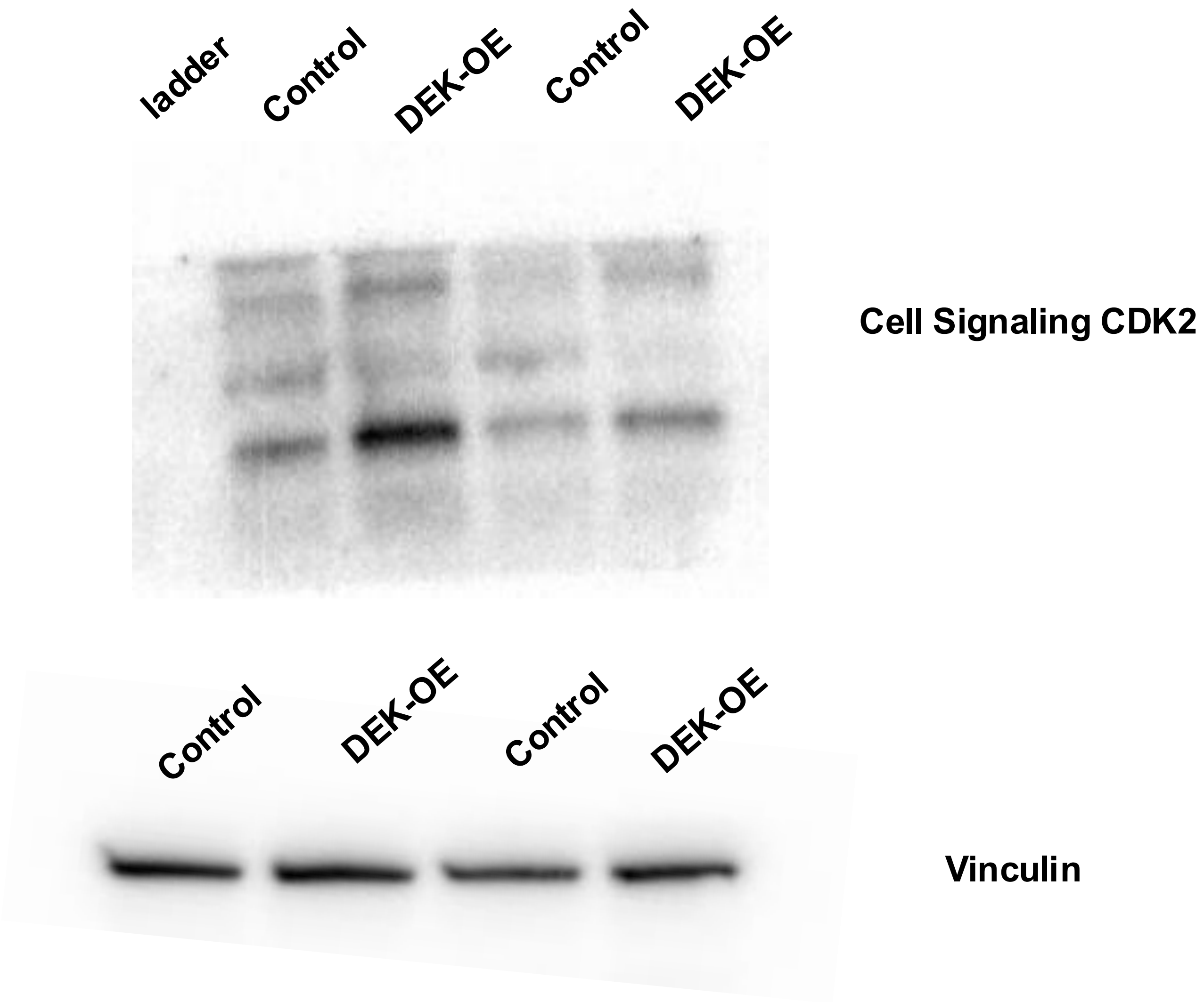
ladder
Control
DEK-OE
Control
DEK-OE
Cell Signaling CDK2
Control
DEK-OE
Control
DEK-OE
Vinculin

Control = +dox

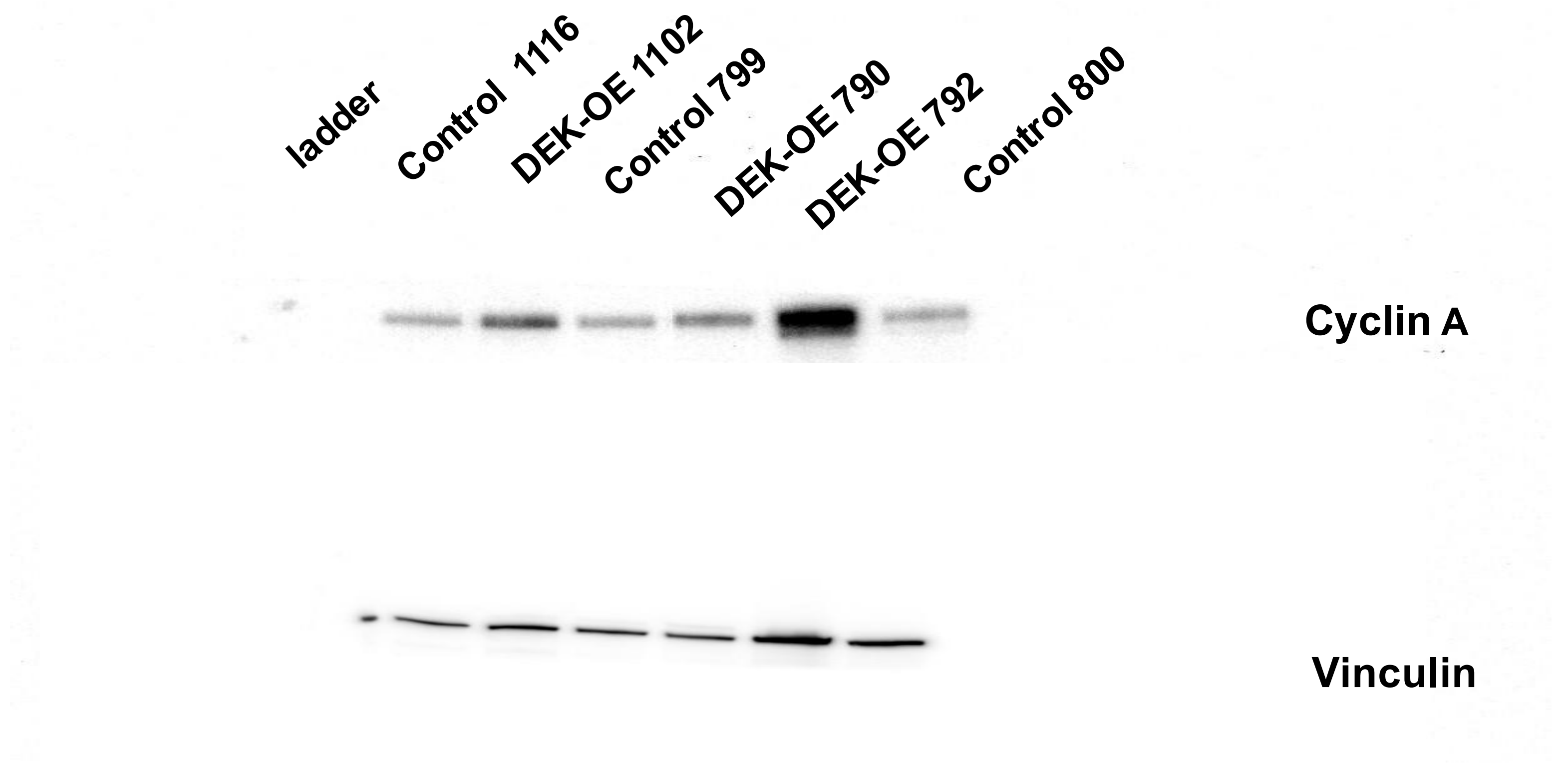
ladder
Control 1116
DEK-OE 1102
Control 799
DEK-OE 790
DEK-OE 792
Control 800
Cyclin A
Vinculin

MG samples

1. 1673 BiLDEK + tTa + **+DOX** (8 weeks)
2. 1687 BiLDEK + tTa (8 weeks)
3. 1674 BiLDEK + tTa + **+DOX** (8 weeks)
4. 1688 BiLDEK + tTa (8 weeks)
5. 1694 BiLDEK + tTa + **+DOX** (8 weeks)
6. 1705 BiLDEK + tTa (8 weeks)
7. 1695 BiLDEK + tTa + **+DOX** (8 weeks)
8. 1706 BiLDEK + tTa (8 weeks)
9. 1696 BiLDEK + tTa + **+DOX** (8 weeks)
10. 1708 BiLDEK + tTa (8 weeks)

Large gel 1

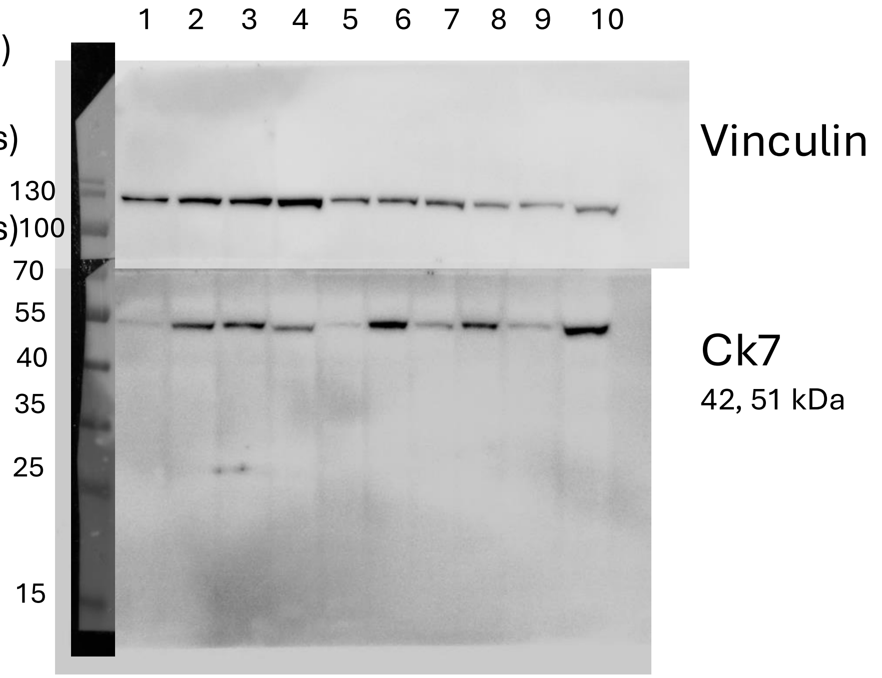

Large gel 2

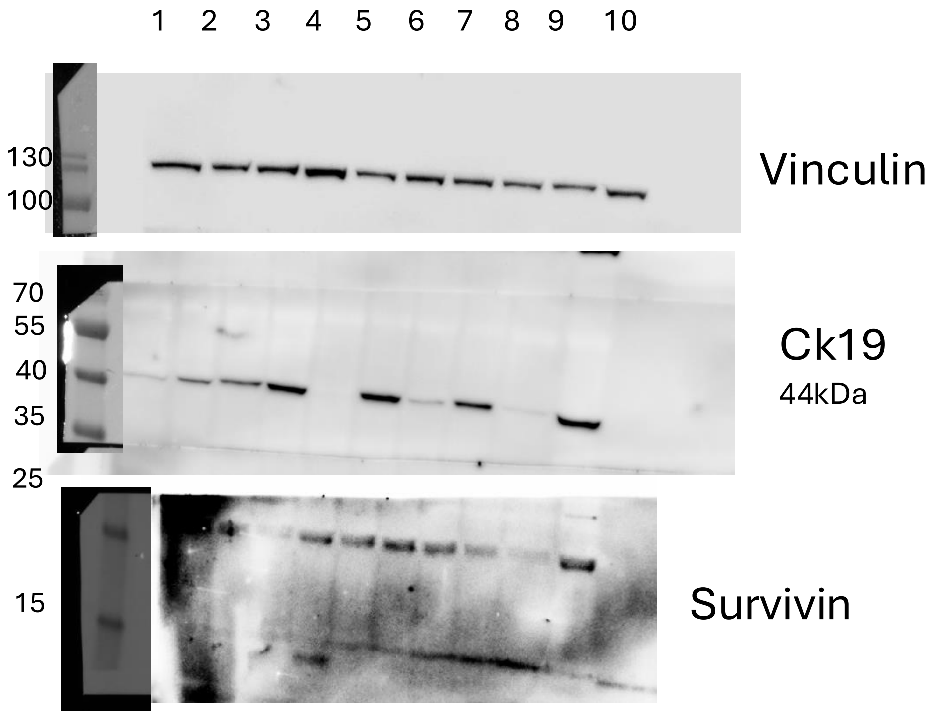

MG samples

1. 1673 BiLDEK + tTa + +DOX (8 weeks)
2. 1674 BiLDEK + tTa + +DOX (8 weeks)
3. 1694 BiLDEK + tTa + +DOX (8 weeks)
4. 1695 BiLDEK + tTa + +DOX (8 weeks)
5. 1687 BiLDEK + tTa (8 weeks)
6. 1688 BiLDEK + tTa (8 weeks)
7. 1705 BiLDEK + tTa (8 weeks)
8. 1706 BiLDEK + tTa (8 weeks)

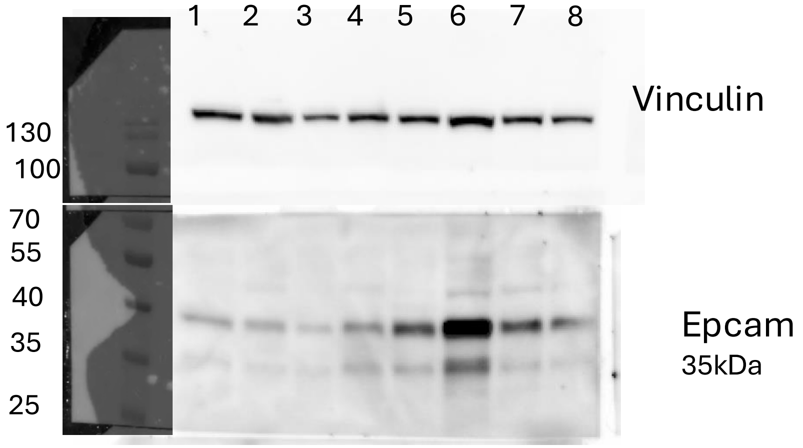

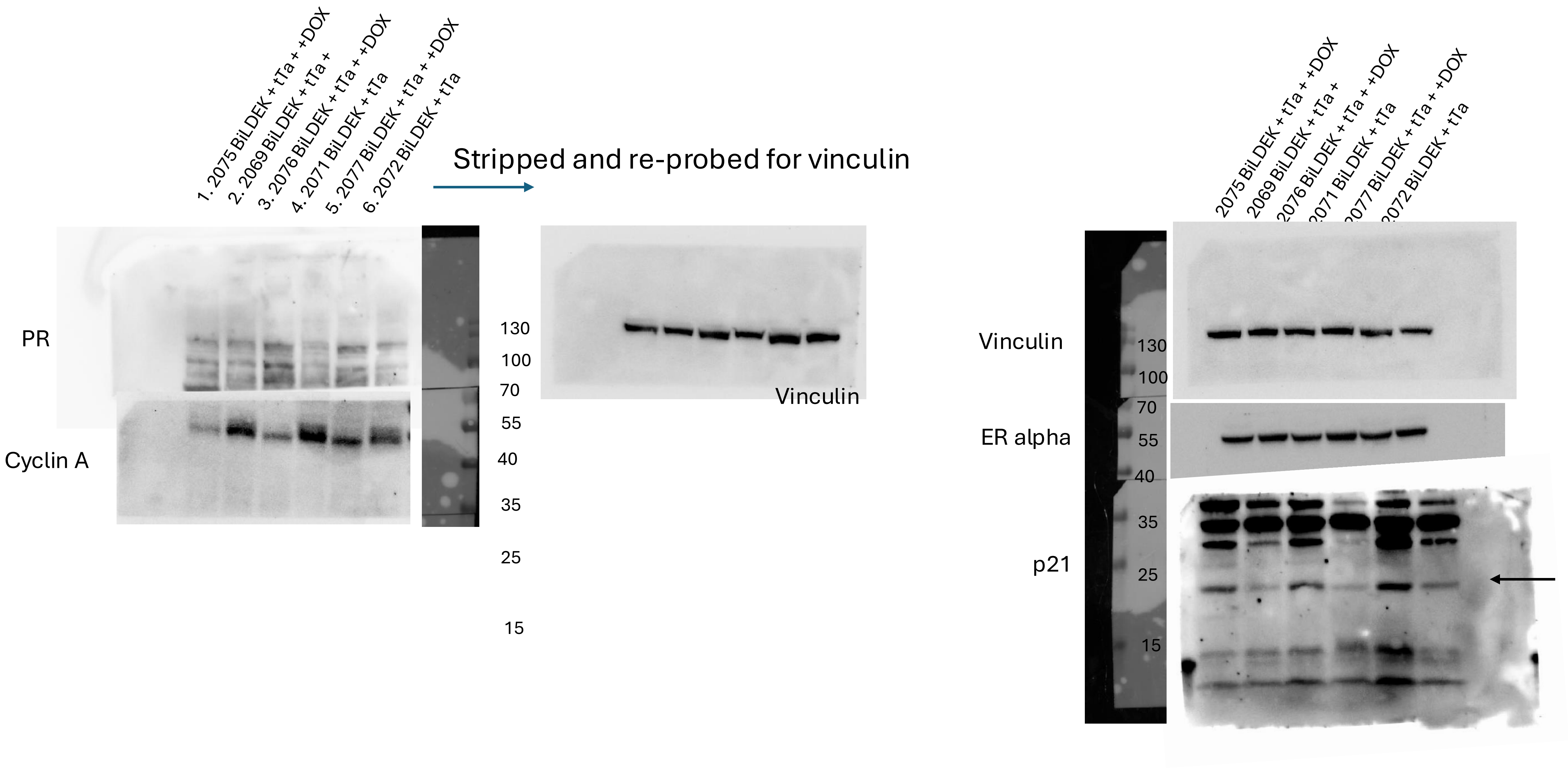

Same samples run on two gels

MCF10A

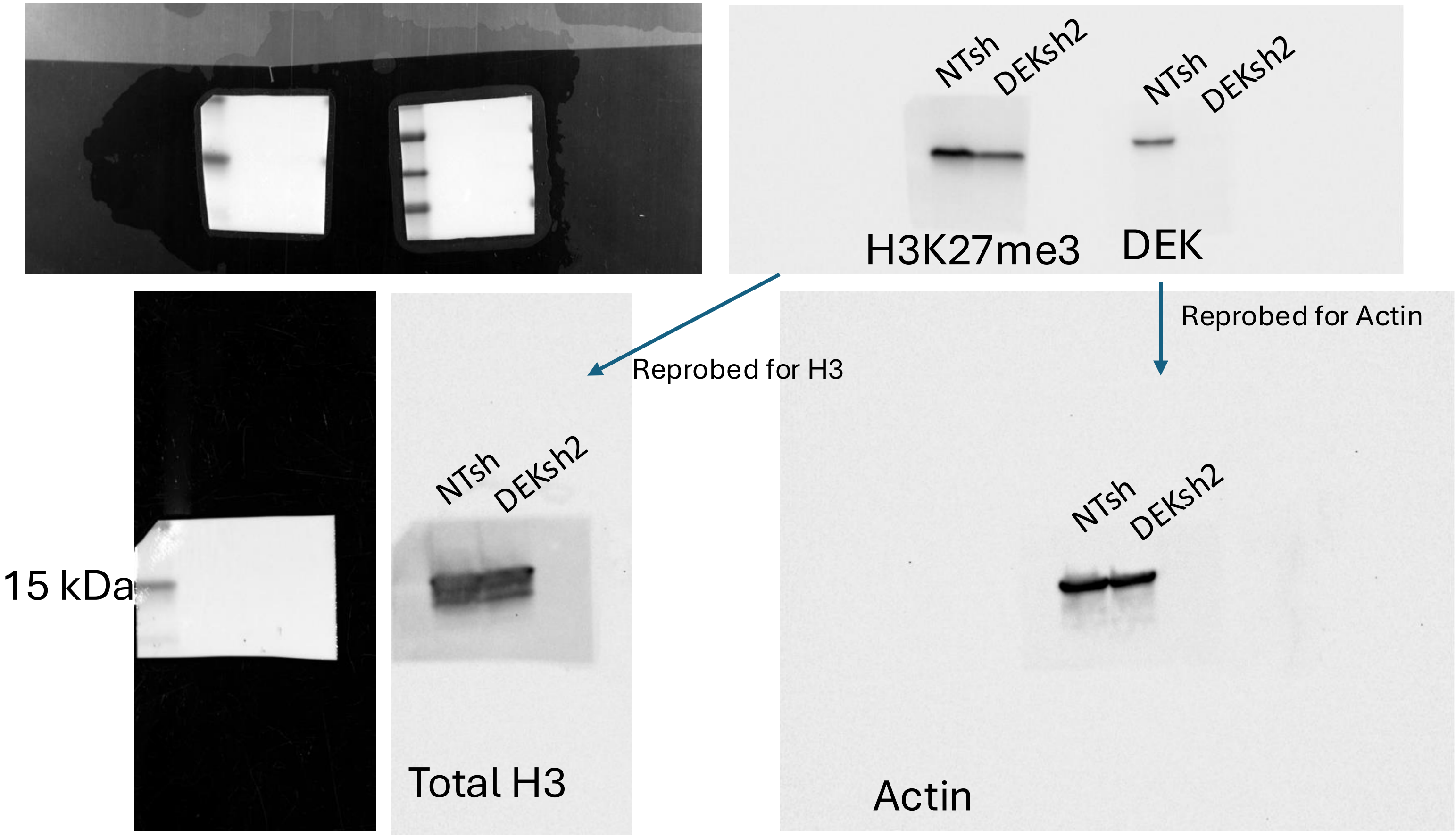
NTsh
DEKsh2
NTsh
DEKsh2
H3K27me3
DEK
Reprobed for H3
Reprobed for Actin
15 kDa
NTsh
DEKsh2
Total H3
NTsh
DEKsh2
Actin

MCF10A pTRIPZ-DEK (* note, only –dox and 2day samples shown in manuscript. 28d cropped out)

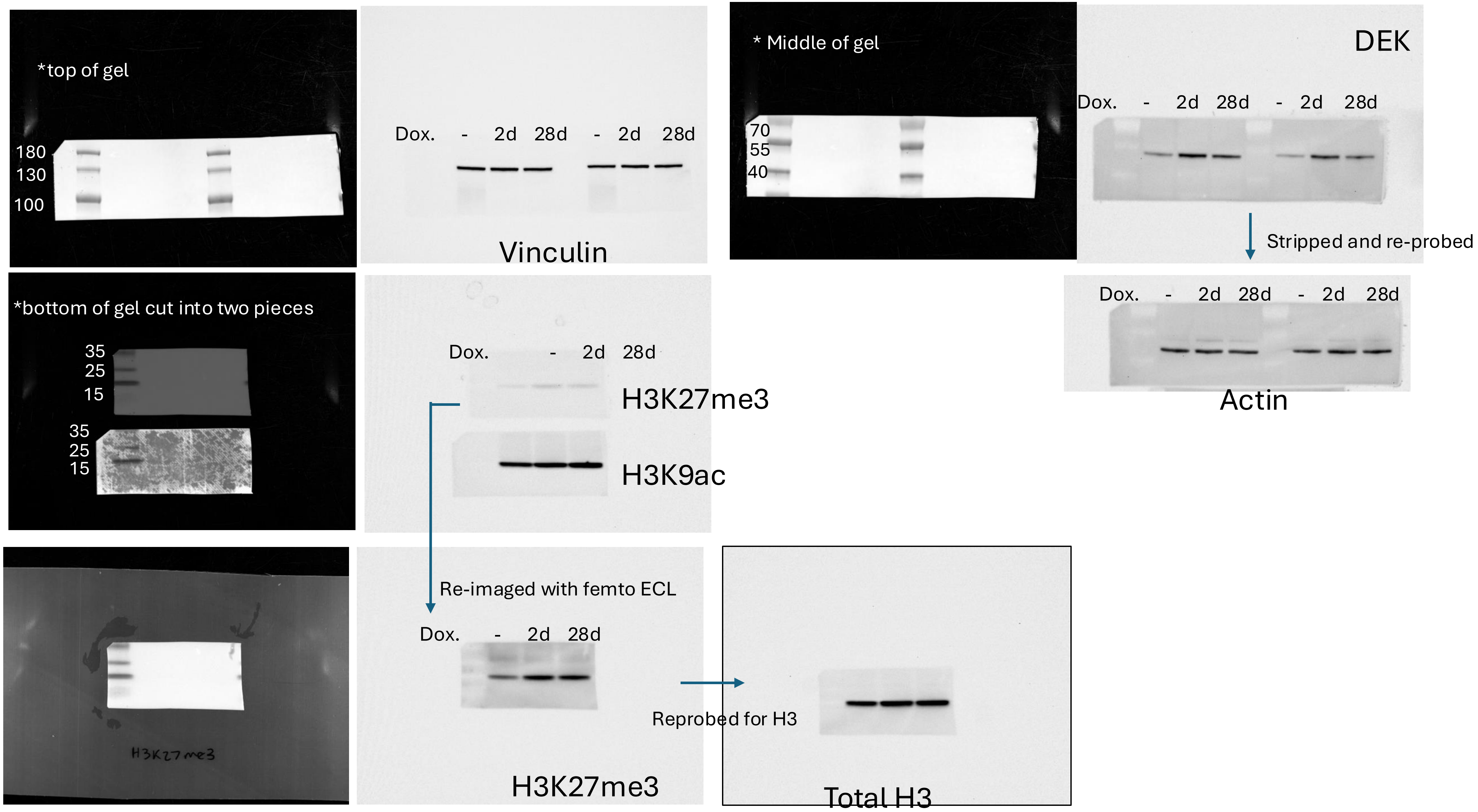

H3 and CK2 input for GFP affinity purification

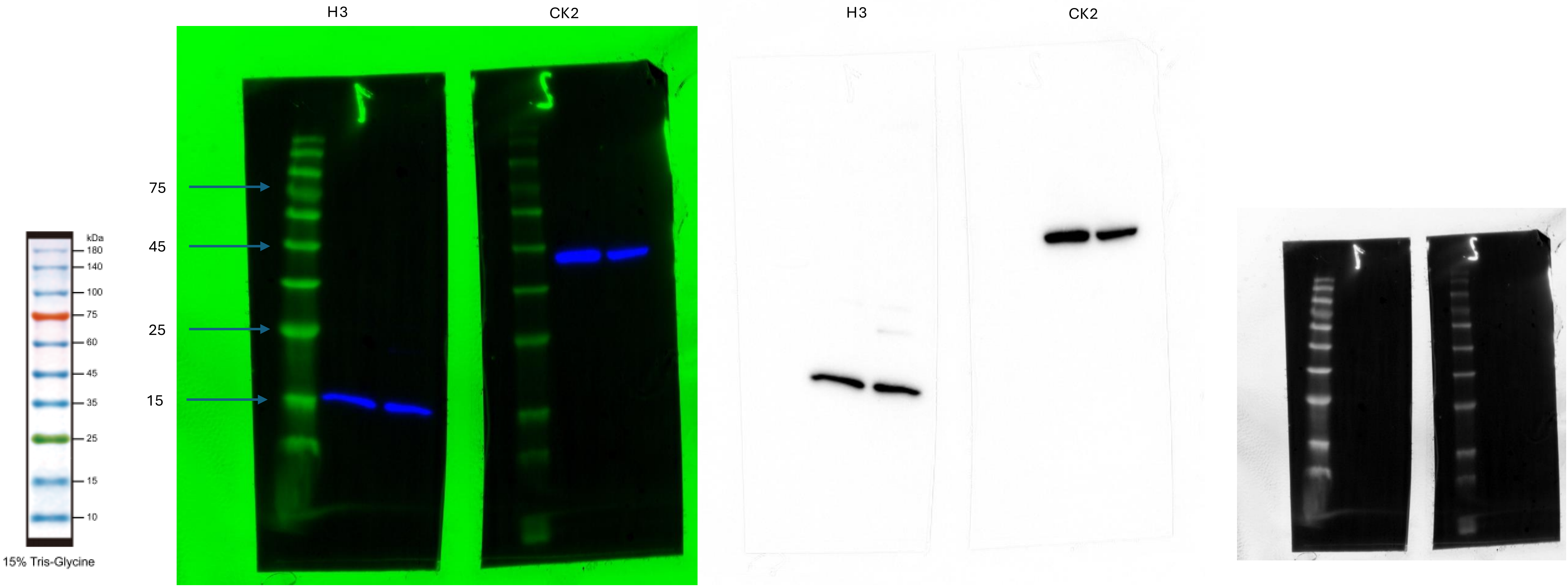

Probes for DEK interacting proteins after GFP affinity purification
With Coomassie and silver stain controls

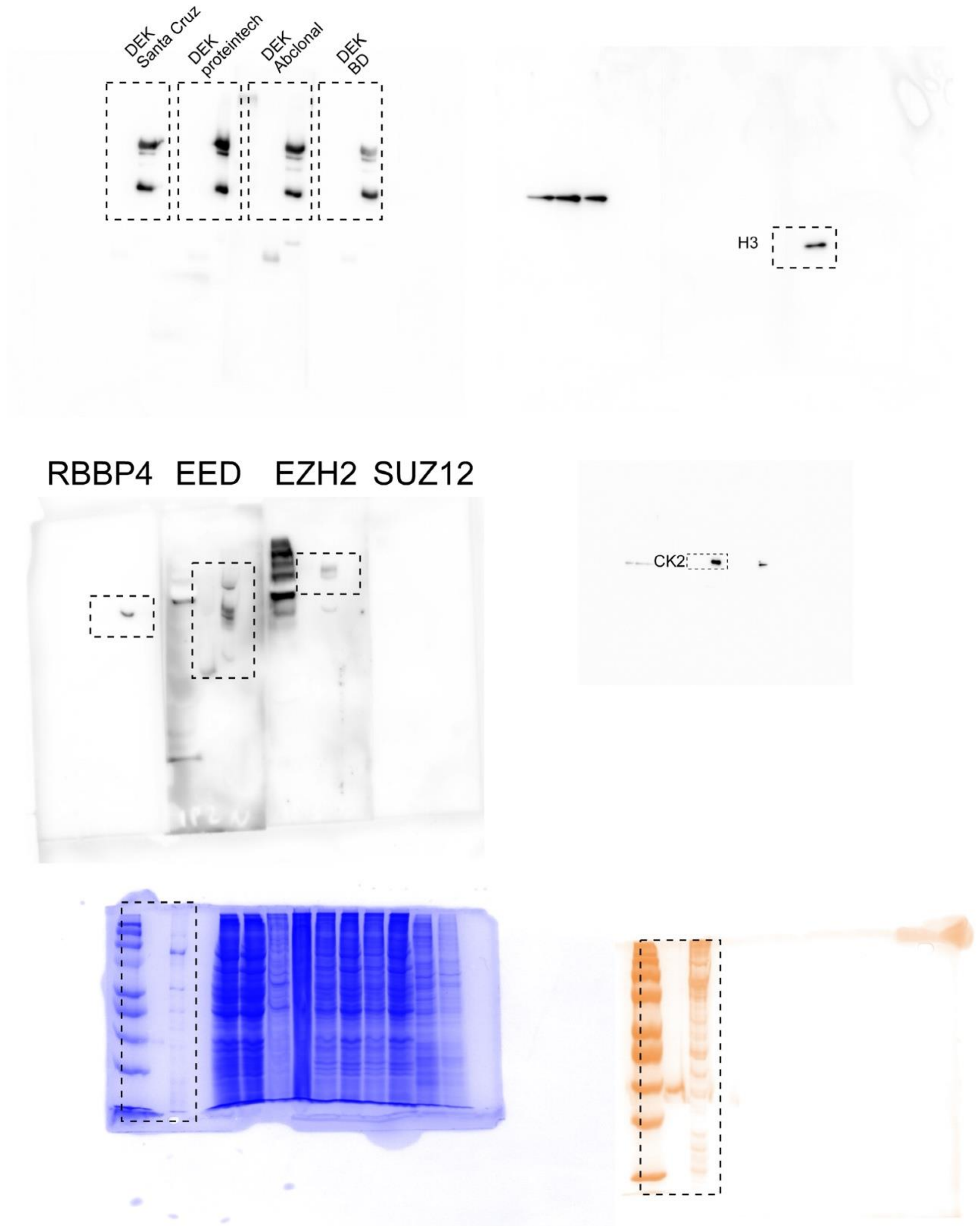

Antibody test: HEK293 WCL (Marker, two identical lanes)

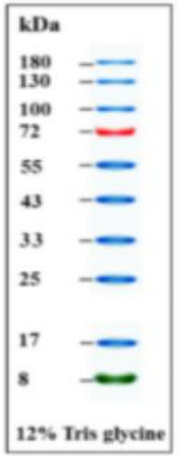

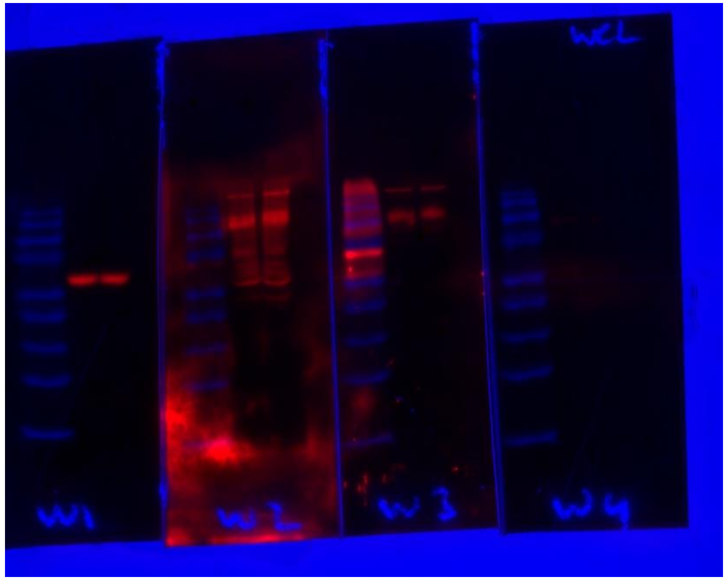

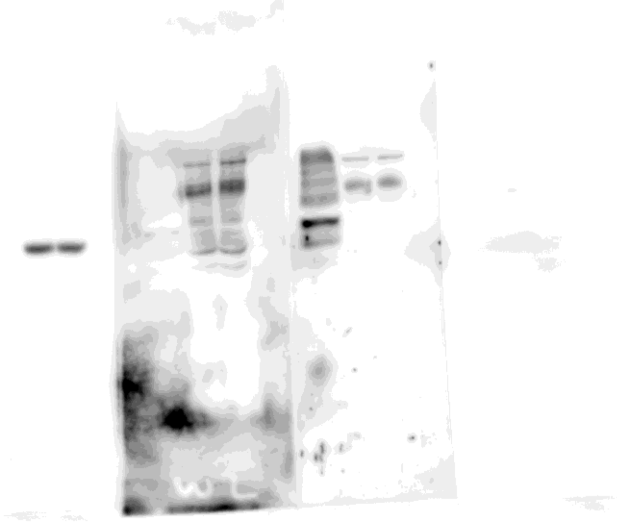

1) RBBP4 2)EED 3)EZH2 4)SUZ12

ABs 1,2 and 3 work
SUZ12 AB is very weak

GFP-trap IP (Marker, GFP-HIS, GFP-HisDEK)

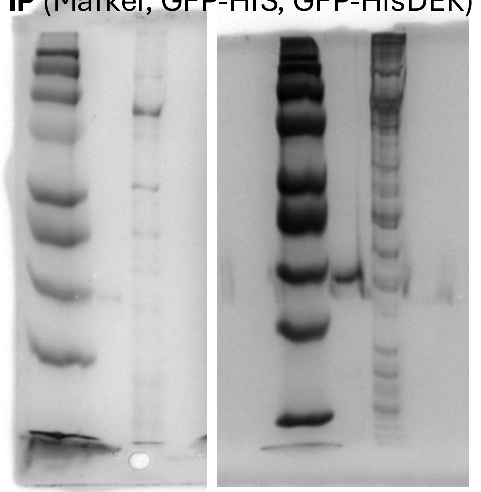

Coomassie Silver

6 ul eluate per lane

100 ul total eluate (7-8 ul per lane)
8 individual strips were probed

GFP-trap IP (Marker, GFP-HIS, GFP-HisDEK)

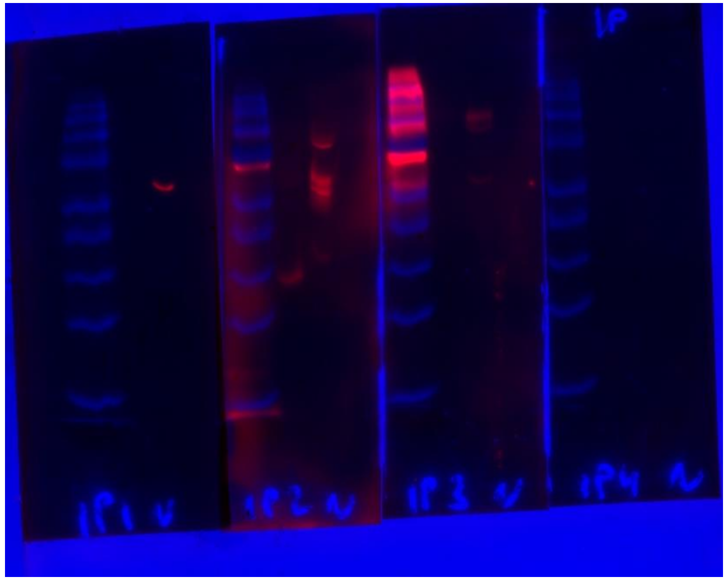

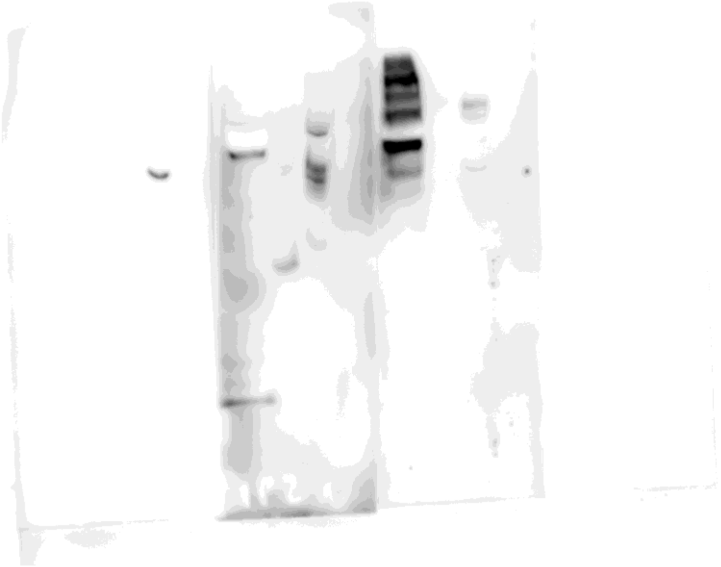

1) RBBP4 2)EED 3)EZH2 4)SUZ12

Clear association of DEK with
RBBP4, EED and EZH2
(no signal with SUZ12, yet weak AB as shown above)

GFP-HIS only lanes are clean

GFP-trap IP (Marker, GFP-HIS, GFP-HisDEK) probed with DEK ABs

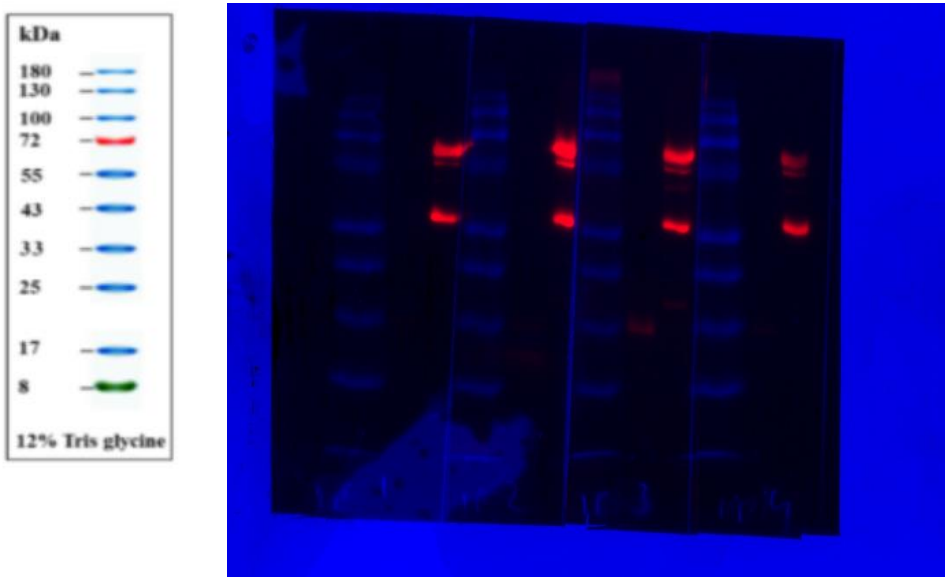

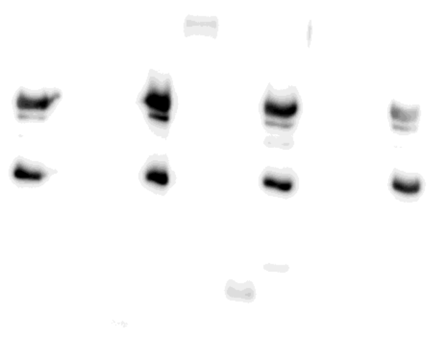

DEK ABs recognize GFP-His-DEK and associated endogenous DEK

GFP-HIS only lanes are clean

1) SC DEK

2)Proteintech DEK

3)Abclonal DEK

4)BD DEK

GFP-trap IP : cytosol, nuclei, input, flow, pellet centrifugation (GFP-HIS, GFP-HisDEK)
Marker , IP GFP-HIS, GFP-HisDEK)

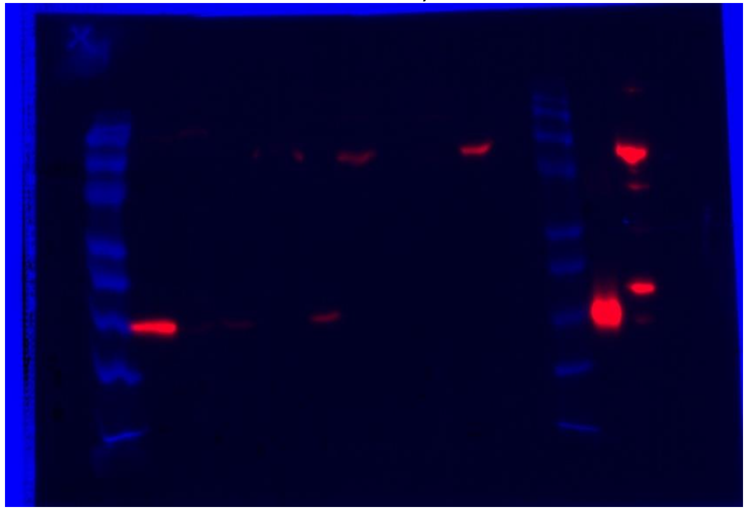

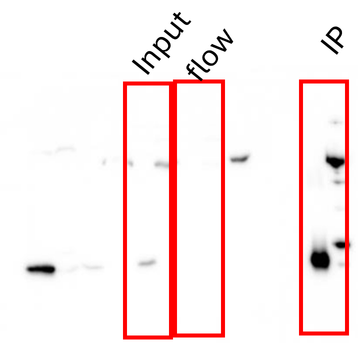

All fractions probed with GFP-AB
Quantitative IP !

GFP-Ab recognized GFP-HIS and GFP-HIS-DEK

Anti-GFP

MCF10A pTRIPZ 885:DEK co-IP

* note: only +Dox samples were shown in manuscript. EZH2/SUZ12 blots developed with Femto ECL

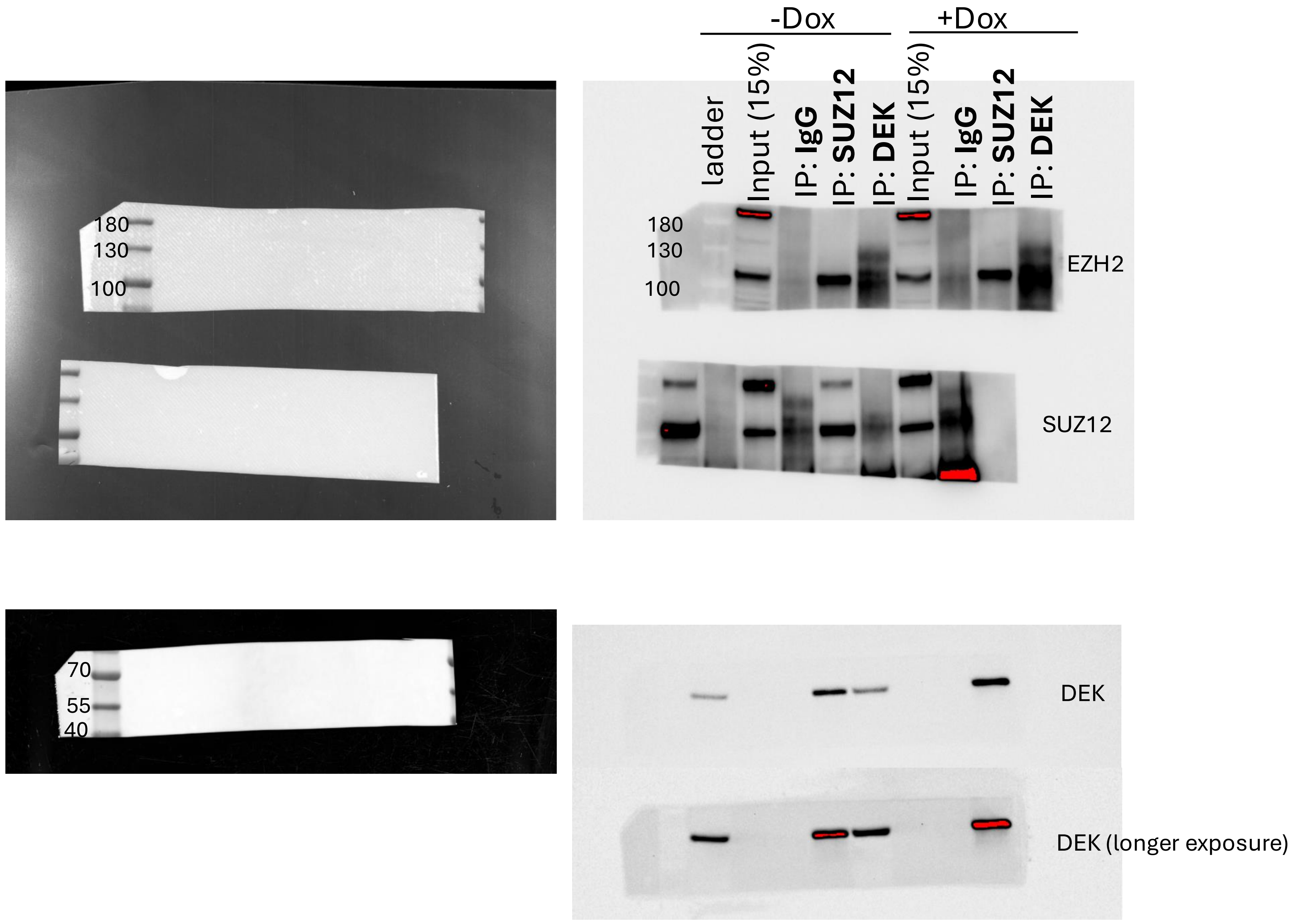

Dek cKO mammary glands
(5 week old mice shown in manuscript)

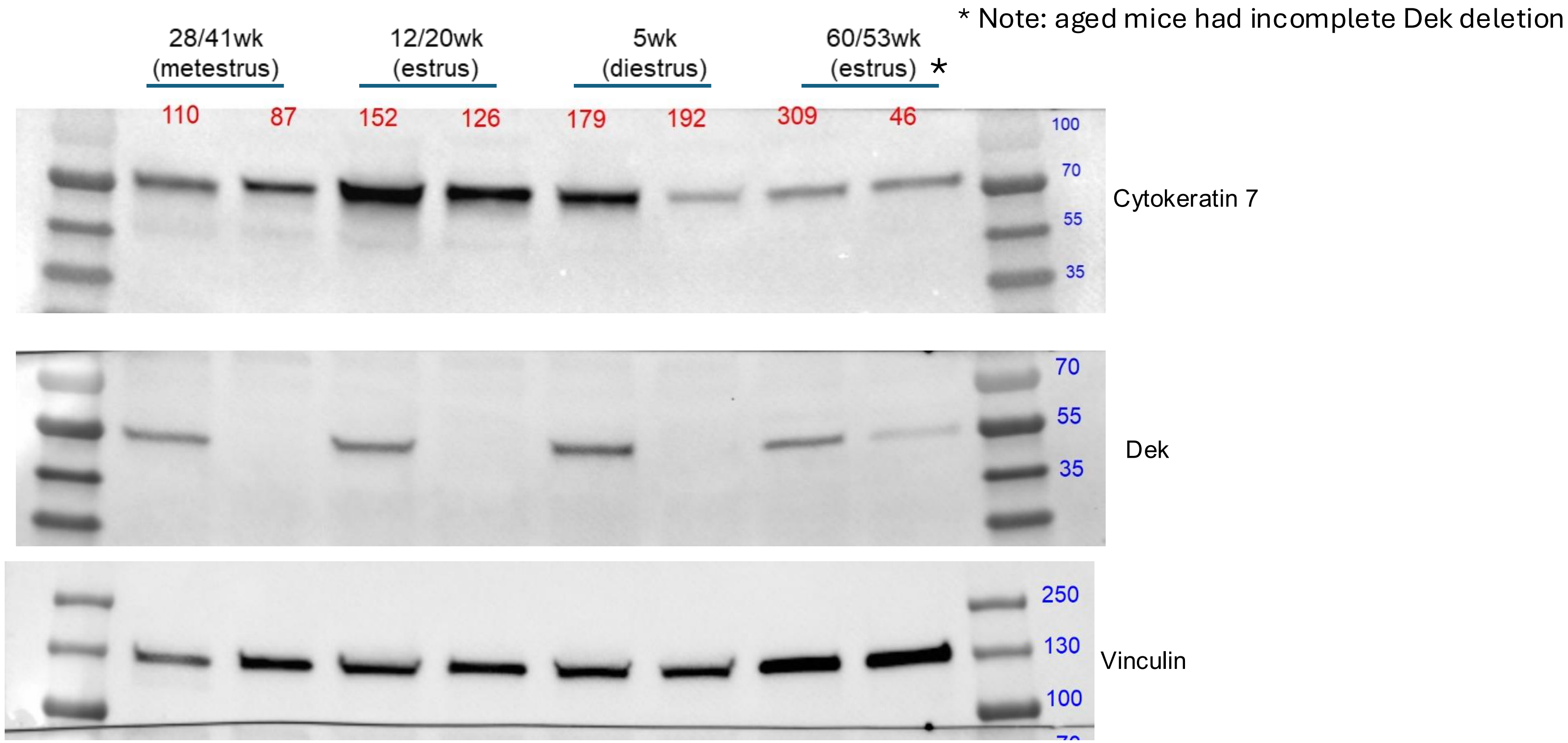

Supplement: Supplementary file 1 [file LSA-2025-03230_SdataF1_F3_F4_F6_F7_FS4.pdf]
